# Preparation and characterization of molecularly imprinted solid-phase extraction column coupled with high-performance liquid chromatography for selective determination of melamine

**DOI:** 10.1098/rsos.180750

**Published:** 2018-09-12

**Authors:** Q. Zhou, X. C. Tan, X. J. Guo, Y. J. Huang, H. Y. Zhai

**Affiliations:** 1 Department of Pharmacy, GuangDong Pharmaceutical University, GuangZhou 510006, People's Republic of China; 2 School of Chemistry and Chemical Engineering, GuangXi University for Nationalities, Nanning 530000, People's Republic of China

**Keywords:** molecularly imprinted, solid-phase extraction, high-performance liquid chromatography, melamine, egg

## Abstract

We synthesized a selective molecularly imprinted solid-phase extraction (MIP-SPE) column and established an extraction and enrichment method using this MIP-SPE column. By coupling with HPLC, we developed a new method to detect trace amounts of melamine in eggs. The MIP-SPE column was synthesized by *in situ* thermal-initiated polymerization using melamine as the template, methacrylic acid as the functional monomer, ethylene glycol dimethacrylate as the cross-linker and azodiisobutyronitrile as the initiator. HPLC was used to evaluate the identification and enrichment capability of the MIP-SPE column and for the measurement of melamine in the sample. The melamine concentration exhibited an excellent linear relationship in the range of 0.1–25.0 µg ml^−1^ (*r* = 0.9983). The identification capability of the MIP-SPE column was apparently superior to that of the non-imprinted polymer solid-phase extraction column; an average enrichment factor of 46.8-fold (RSD = 3.5%) was obtained for 0.4 µg ml^−1^ melamine by the MIP-SPE column. When the MIP-SPE HPLC method was applied to the detection of melamine in eggs, an average recovery rate of 93.5–102.0% (RSD = 3.6–4.9%) and a limit of detection of 0.05 µg kg^−1^ were obtained. This method is simple, fast and cost-effective; thus, it can greatly simplify the pre-treatment of complex samples and can be used in the detection of residual melamine in eggs and other products.

## 1. Introduction

**Scheme 1.**
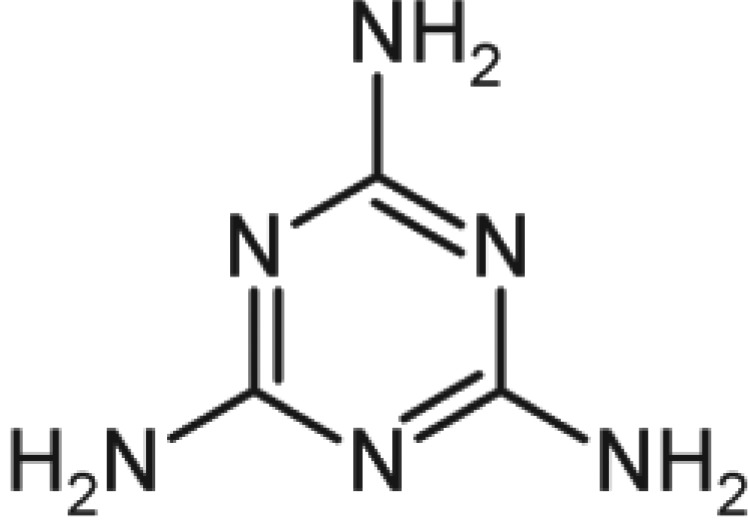
Chemical structure of melamine.

Melamine is a nitrogen heterocyclic compound (Scheme 1), whose IUPAC name is 1,3,5-triazine-2,4,6-triamine. As an important chemical raw material, melamine is widely used in the coating, wood processing, textile, leather and medical industries [1]. However, melamine is not a food additive and cannot be added in food products.

As the nitrogen content in melamine is as high as 66%, some illegal merchants add it to food products to improve the protein content. Melamine is therefore commonly called ‘protein essence' in China. The adulteration of milk with melamine was the culprit in the 2008 Chinese milk scandal (or the Sanlu milk scandal), leading to kidney stones in young children. Therefore, it is extremely important to establish a simple, rapid and low-cost method for the detection of trace amounts of melamine in food products.

Currently, the main detection methods for melamine in food products include high-performance liquid chromatography (HPLC) [2–9], liquid chromatography–mass spectrometry (LC-MS) [10–14], gas chromatography–mass spectrometry (GC-MS) [15–18], capillary electrophoresis (CE) [19–21] and mass spectrometry (MS) [22]. However, these methods share the common flaw of requiring complicated sample pre-treatment. Almost all the methods reported in the references need to go through the steps of solvent extraction, centrifugation separation and filtration. Some references also added steps for purification by the C18-SPE column. Zhu *et al*. [3] synthesized a molecularly imprinted stir bar for the enrichment of melamine. The sample recovery of this method is not high. Zhang *et al*. [7] used a Sil-g-P(SPM/EDMA)-g-pTMMA column to purify and enrich melamine. This column has no molecular recognition capability. Liu *et al*. [8] synthesized molecularly imprinted polymer (MIP) powder for the enrichment of melamine, but the process was complex and takes a long time.

Solid-phase extraction (SPE) uses simple equipment, and it affords simplification, high efficiency and flexibility to the sample treatment, resulting in rapid progress in improving tedious and complex sample treatment technologies [23]. MIP possesses an excellent stereospecific identification capability and has been widely used in chromatographic separation, the simulation of antibodies or antigens, biosensors and the simulation of enzymes and catalytic synthesis [24,25]. Owing to the specific identification and excellent adsorption capability, the MIP-SPE column synthesized for the detection of melamine can be used for the *in situ* separation and enrichment of trace amounts of melamine in eggs, which can be detected by HPLC. This method resolves the selective analysis problem inherent with low concentrations of the detected components and complex sample systems.

## 2. Experimental

### 2.1. Equipment and reagents

An SHZ-D(III) circulating water vacuum (Yu Hua Instrument), a DA-3A ultrasonic extractor (Lingtong Electronics, Lecong, Shunde) and an LSP01-1A microsyringe pump (Longer Pump) were used. The Agilent 1200 high-performance liquid chromatograph includes a G1311A quaternary pump, G1316A thermostatted column, G1322A vacuum degasser, G1314B variable wavelength detector and G1328B manual injector (20 µl). The S-520 scanning electron microscope (SEM) is produced by Hitachi.

Melamine, ethylene glycol dimethacrylate (EDMA), methacrylic acid (MAA), azodiisobutyronitrile (AIBN) and other analytical reagents were purchased from the Aladdin Reagent Company (Shanghai, China). HPLC-grade methanol was supplied by Merck (chromatographically pure, Germany). Glass capillary (10 cm long, 500 µm i.d.) was purchased from the West China Center of Medical Sciences (China). Benzene, 1-dodecanol and other analytical reagents were purchased from the Guangzhou Chemical Reagents Factory (China), and eggs were purchased from local markets. After ultrasonic degassing and filtration through 0.22 µm membranes, all solutions were used for HPLC analysis.

### 2.2. Preparation of the melamine molecularly imprinted solid-phase extraction column

A total of 0.0085 g of the template molecule melamine (0.067 mmol) was weighed and placed in a 25 ml conical flask. After adding 0.04 ml (0.4 mmol) of MAA, 2 ml of methanol and 400 µl of water, the solution was sonicated for 30 s for uniform mixing and sufficient reaction [26–28]. Then, benzene and 1-dodecanol (0.5/1.5 ml) were added, and the mixture solution was sonicated for 1 min. Finally, 1 ml of EDMA and 10 mg of AIBN were added. The mixed solution was then purged with N_2_ for 1 min and siphoned to the glass capillary column, with both ends sealed by rubber stoppers. Subsequently, polymerization was initiated at 75°C in a water bath for 5 h. The capillary column was then washed by a microsyringe pump with methanol–ammonia (7 : 3, v : v) to remove redundant templates and unreacted reagents. Finally, methanol (500 µl, 1 ml h^−1^) was used to equilibrate the column, which was stored and ready for use after blow-drying with N_2_.

SEM was used to characterize the morphology of the MIP-SPE column at a magnification from 1000 to 20 000.

The corresponding blank non-imprinted polymer (NIP)-SPE column was prepared in the same manner in the absence of template.

### 2.3. Selection of eluents

**Figure 1.**
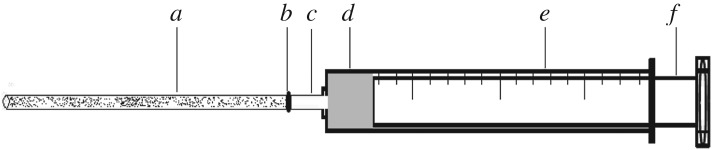
Loading of sample, enrichment and elution set-up of the MIP-SPE column: (*a*) SPE column, (*b*) connection, (*c*) front-end of needle, (*d*) solution cavity, (*e*) needle body and (*f*) hand propeller.

Methanol–acetic acid, methanol–ammonia and pure methanol of different polarities were selected as the eluents for the elution of melamine. When pure methanol was used as the eluent, the recovery rate of template molecules was less than 30%; when methanol–acetic acid (9 : 1, v : v) was used as the eluent, the recovery rate of template molecules was 60%; and when methanol–ammonia (7 : 3, v : v) was used as the eluent, the recovery rate was greater than 90%. Therefore, methanol–ammonia (7 : 3, v : v) was selected as the eluent in this experiment. Figure 1 shows the set-up, in which the microsyringe pump provides thrust for fluids to flow through the MIP-SPE column.

### 2.4. Solution preparation

A total of 0.0126 g of melamine control sample was sonicated, so it would dissolve in methanol–water (5 : 1, v : v) and brought to a constant volume in a volumetric flask (25 ml, 25°C). The 0.5 mg ml^−1^ stock solution was thus prepared and stored in a 4°C refrigerator. Methanol–water (5 : 1) was used to dilute the stock solution and prepare 0.1, 1.0, 2.0, 5.0, 10.0, 15.0 and 25.0 µg ml^−1^ melamine standard solutions for plotting standard curves.

Standard solutions of other concentrations were prepared by diluting the stock solution when needed.

### 2.5. Enrichment factor

After the MIP-SPE column was activated by methanol–water (5 : 1), the column was air-dried. Then, the injection pump was used to inject 1 ml of the sample solution with a concentration of 0.4 µg ml^−1^ by setting the flow rate at 1 ml h^−1^. Next, 20 µl of methanol–ammonia (7 : 3) eluent was used to elute the column. The eluent was collected, evaporated and brought to a volume of 200 µl. The experiments were repeated three times. After filtration, the concentration of the eluent was determined by HPLC.

The enrichment factor refers to the concentration (*C*_i_) of the components adsorbed and retained on the column, which was eluted by a small volume (*V*_1_) of eluent and whose concentration was measured from the eluent when a sample with a certain concentration (*C*_0_) and volume (*V*_0_) flowed through the MIP-SPE column. The enrichment factor (*N*) is the ratio of the concentration of this component after and before adsorption and retention, i.e. *N* = *C_i_*/*C*_0_. The set-up is shown in figure 1.

### 2.6. Sample pre-treatment

Eggs were crushed and stored in beakers. Glass rods were used to mix egg yolk and egg white. Then, 1.0 g of the egg mixture, 0.5 ml of water, 3.5 ml of acetonitrile and 3.5 ml of methanol were mixed and vortex-oscillated for 1 min, sonicated for 20 min and centrifuged for 10 min (10 000 r.p.m.). The supernatant liquid was transferred to a beaker. n-Hexane (5 ml) was used for extraction, and the n-hexane layer was removed. The subnatant was concentrated at 75°C to near dryness. Methanol–water (5 : 1, v : v) was added to bring the volume to a constant in a 5 ml volumetric flask. After flowing though the custom-made melamine MIP-SPE column for enrichment and elution, the solution was filtered through a 0.22 µm membrane and ready for measurement.

In the determination of the recovery rate, a predetermined amount of melamine standard solution was added to the quantitatively weighed egg sample. The other steps were the same.

### 2.7. High-performance liquid chromatography conditions

C18 columns (ZORBAX Eclipse XDB, 250 mm × 5 µm) were purchased from Agilent, and the following conditions were used: mobile phase, methanol–water (40 : 60, v : v); UV detection wavelength, 240 nm; flow rate, 0.8 ml min^−1^; and thermostatted column, 20°C.

## 3. Results and discussion

### 3.1. Scanning electron microscope images of the melamine molecularly imprinted solid-phase extraction column

**Figure 2.**
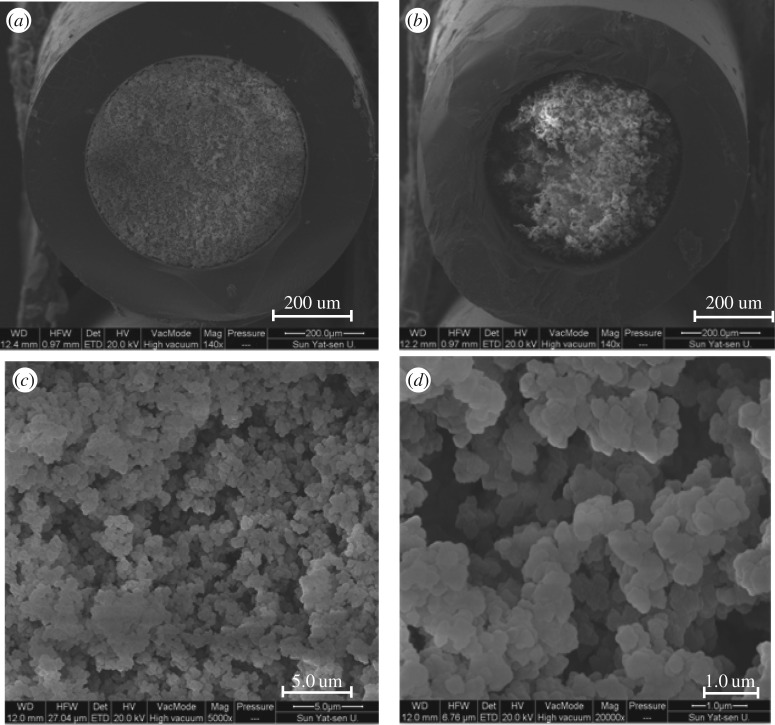
SEM of the cross-section of the MIP-SPE column. (*a*) Cross-section of the MIP-SPE column with successful polymerization. (*b*) Cross-section of the MIP-SPE column with failed polymerization. (*c*) Cross-section of the MIP-SPE column at a magnification of 5000. (*d*) Cross-section of the MIP-SPE column at a magnification of 20 000.

SEM was used to characterize the morphology of the MIP-SPE column (figure 2). The results show a strong binding between the inner wall of the glass capillary and the polymer, indicating that the mechanical strength of the column is high. The imprinted polymer particles exhibited uniform size and regular shape. In addition, dense pores existed between the particles, indicating that the column may have a strong adsorption capacity, excellent permeability and excellent flow characteristics.

### 3.2. Standard curve and detection limit

The melamine concentration was measured using a MIP-SPE column HPLC based on the chromatographic conditions described in §2.4, and a linear equation of *y* = 11.559x + 0.4942 (*r* = 0.9983) for the standard curve was obtained, indicating that the melamine standard solution exhibited an excellent linear relationship in the concentration range of 0.1–25.0 µg ml^−1^. The detection limit was 0.05 µg ml^−1^ (25 ng kg^−1^, S/N = 3). If the measurement was performed after enrichment, the detection limit further decreased to 0.5 µg kg^−1^ (1 ng ml^−1^). The maximum amount of melamine allowed in infant formula is 1 mg kg^−1^ in China, Australia and Canada, while the limit is 0.25 mg kg^−1^ for dairy products in the USA. Therefore, the method in this paper meets the requirement for a detection limit.

### 3.3. Characterization of the properties of the molecularly imprinted solid-phase extraction column

#### 3.3.1. Breakthrough curve and column capacity

**Figure 3.**
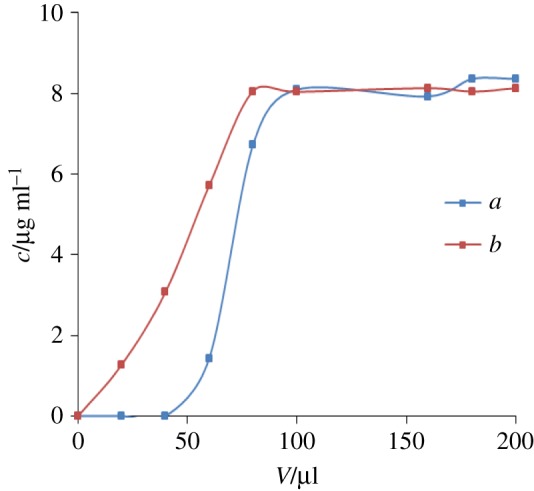
Breakthrough curves of 10.0 μg ml^−1^ melamine standard solution by (*a*) a MIP-SPE column and (*b*) a NIP-SPE column.

After the MIP-SPE column was activated by 20 µl of methanol–water (5 : 1), 10.0 µg ml^−1^ melamine standard solution was loaded onto the column with a loading volume of 20 µl and a total volume of 200 µl. The eluent was brought to a volume of 200 µl and measured by HPLC after filtration through a membrane. A plot (figure 3) was obtained by using the loading volume as the abscissa and the effluent concentration as the ordinate. The figure indicates that the recognition capability of melamine by the MIP-SPE column is apparently superior to that by the NIP-SPE column.

The maximum column capacity is calculated by the difference between standard solution concentration and eluent concentration. The calculation shows that the maximum column capacity of the MIP-SPE column is 0.1779 mg · g^−1^, while that of the NIP-SPE column is 0.05988 mg · g^−1^. Thus, the MIP-SPE column has a larger column capacity, indicating that the imprinting efficiency is high and it can meet the measurement requirements of different sample volumes.

#### 3.3.2. Enrichment factor

**Figure 4.**
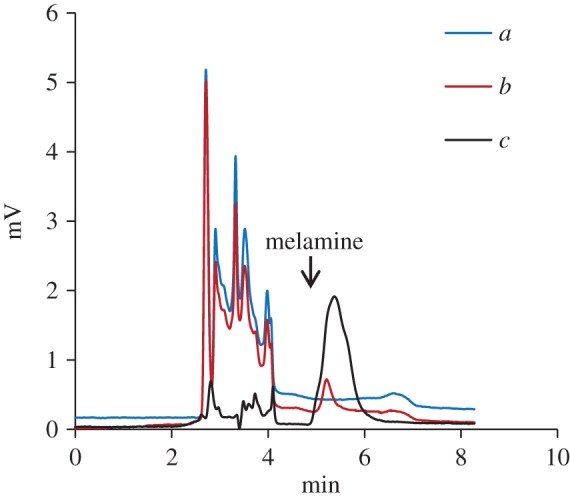
HPLC chromatograms of blank (*a*) and the egg samples before enrichment (*b*) as well as after enrichment (*c*) by the MIP-SPE column.

The experiment and calculations were performed based on the method described in §2.5, and the results are shown in table 1 and figure 3. The MIP-SPE column has an average enrichment factor of 46.8 for melamine, exhibiting an excellent enrichment effect. Figure 4*c* shows that the MIP-SPE column also exhibits excellent impurity removal efficiency.

**Table 1.** Determination of enrichment factor by the MIP-SPE column for melamine.

no.	initial concentration *C*_0_(μg ml^−1^)	concentration after enrichment *C_i_*(μg ml^−1^)	enrichment factor	average enrichment factor	RSD/%
1	0.4	18.01	45.0	46.8	3.5
2	0.4	18.87	47.2		
3	0.4	19.27	48.2		

### 3.4. Sample analysis

Two batches of eggs purchased from markets were treated based on the methods described in §2.6 and measured by HPLC after enrichment and elution by the MIP-SPE column. The measured melamine concentrations were 0 and 0.1001 µg ml^−1^. The recovery experimental results are shown in table 2; the average recovery rate was in the range of 93.5–102.0%, while the RSD was in the range of 3.6–4.9%.

**Table 2.** Application of MIP-SPE HPLC for the detection of melamine in eggs (*n* = 5).

samples	found (µg ml^−1^)	added (µg ml^−1^)	determined (µg ml^−1^)	recovery, *n* = 5 (%)	RSD, *n* = 5 (%)
1	0	0.1	0.1023	102.0	3.6
2	0	0.3	0.2825	93.7	4.9
3	0	0.7	0.6744	96.3	4.4
4	0.1001	0.1	0.1987	98.7	4.1
5	0.1001	0.3	0.3838	94.3	4.6
6	0.1001	0.7	0.7545	93.5	4.7

### 3.5. Comparison of this method with methods in the literature

Compared with the HPLC, LC-MS, GC-MS, CE and other methods in the literature, C18-SPE is generally used for pre-treatment of samples, while in this study, we used MIP-SPE synthesized by ourselves, MIP-SPE column has the characteristics of strong enrichment ability and selective recognition ability, the recovery rate is higher than those of LC-MS and GC-MS, and the detection limit is comparable to those of LC-MS and GC-MS but apparently better than that of HPLC (table 3).

**Table 3.** Comparison of this method with references for melamine detection.

ref.	method	linear range	recovery (%)	LOD	RSD (%)	real samples
[2]	HPLC	7–8000 ng ml^−1^	85–95	2.0–5.8 ng ml^−1^	3.9–6.6	dairy products
[4]	HPLC	1–8 µg ml^−1^	97.2–101.2	0.1 µg ml^−1^	<1.0	infant formula
[12]	LC-MS	50–1000 pg µl^−1^	83–102.5	600 ng l^−1^	6.5	milk powder
[14]	LC-MS	5–500 µg ml^−1^	92.0–97.9 86.9–88.6	0.2 µg kg^−1^ 1.3 µg kg^−1^	3.5–6.8 6.4–9.6	soil, strawberry
[15]	GC-MS	50–800 ng ml^−1^	80.8–101.5	0.02 mg kg^−1^	3.6–7.9	milk powder
[17]	GC-MS	0.001–1000 µg ml^−1^	64.9–105.9	0.001 µg ml^−1^	2.8–9.3	dairy products
[19]	CE	6.25–100 µg ml^−1^	92.13–102.47	4 mg kg^−1^	2.07–4.98	milk powder
	proposed method	0.1–25.0 µg ml^−1^	93.5∼102.0	0.5 µg kg^−1^ (1 ng ml^−1^)	3.6–4.9	egg

## 4. Conclusion

We synthesized a selective molecularly imprinted solid-phase extraction (MIP-SPE) column that has a specific identification capability for melamine. By coupling with HPLC, we successfully detected trace amounts of melamine in eggs purchased from markets. The results showed that the MIP-SPE column has a better identification capability for melamine than the NIP-SPE column; the maximum column capacity of the MIP-SPE column is 0.1779 mg g^−1^; the MIP-SPE column has an average enrichment factor of 46.8 for melamine; the melamine standard solution exhibits an excellent linear relationship in the concentration range of 0.1–25.0 µg ml^−1^; the detection limit is 0.5 µg kg^−1^ if the measurement is performed after enrichment; and the average recovery rate is in the range of 93.5–102.0%. The MIP-SPE column has advantages such as high selectivity, strong anti-interference capability, fast and simple sample treatment and cost-effectiveness. By coupling with HPLC or other analytical methods, this method can be used for the detection of trace amounts of melamine in food products.

## Data accessibility

All data are included in the article.
